# Sparse Coding and Temporal Pattern Learning Co-Mediated by Dual Spike-Timing-Dependent Plasticity in a Multilayer Excitatory–Inhibitory Spiking Network

**DOI:** 10.3390/biomimetics11070462

**Published:** 2026-07-02

**Authors:** Chunhua Yuan, Deyang Wang, Xiangyu Li, Xianwen Gao

**Affiliations:** 1 School of Automation and Electrical Engineering, Shenyang Ligong University, Shenyang 110159, China; yuanch@sylu.edu.cn (C.Y.); 2406620717@stu.sylu.edu.cn (D.W.); 2 College of Information Science and Engineering, Northeastern University, Shenyang 110819, China; gxwneu@163.com

**Keywords:** spiking neural network, spike-timing-dependent plasticity, excitatory–inhibitory balance, sparse coding, winner-take-all, mutual information, neuromorphic computing

## Abstract

Excitatory–inhibitory (E-I) local circuits play a central role in synaptic plasticity and neural coding, yet their multilayer learning dynamics remain poorly understood. We constructed a multilayer feedforward spiking neural network with intra-layer E-I connectivity, using Izhikevich neurons to model regular spiking (RS) and fast spiking (FS) cells, and examined cooperative learning under excitatory and inhibitory spike-timing-dependent plasticity (eSTDP and iSTDP). FS-mediated lateral inhibition alleviates the long-term depression bias arising from RS firing rate adaptation via winner-take-all competition, promoting heterogeneous E→E weight differentiation while preserving mean synaptic strength. A 12×12 parameter grid scan shows that iSTDP expands the stable learning region in the E-I parameter space and reveals a sustained cooperative co-evolution of eSTDP and iSTDP during training. For sparse coding, RS adaptation is the primary driver of Lifetime Sparseness, with FS inhibition acting as a cooperative enhancer; the network exhibits low sparseness at the input layer, a rapid increase at the second layer, and a stable plateau in deeper layers. For temporal pattern learning, the selectivity index $d^{\prime }$ improved substantially after training, reaching approximately 1.90 times that of the FS-absent condition; both interval sensitivity and pattern generalization tests confirmed that this advantage is robust across biologically plausible inter-group delays and preserved under small temporal jitter. Mutual information analysis reveals a consistent tendency for intra-layer FS circuits to maintain higher stimulus-related information across deep layers, consistent with FS-mediated suppression of non-specific responses. These findings provide computational evidence, within the scope of the present model, for understanding cortical E-I cooperative plasticity and inform design principles for neuromorphic systems with adaptive inhibitory regulation.

## 1. Introduction

The cerebral cortex processes information through the coordinated activity of excitatory and inhibitory (E-I) neurons, where the precise temporal structure embedded in spike activity serves as a fundamental substrate for neural coding and learning [1]. Disruption of E-I balance has been implicated in neuropsychiatric disorders including epilepsy and autism spectrum disorder [2], highlighting the functional importance of inhibitory regulation in cortical circuits. Inspired by these biological principles, spiking neural networks (SNNs), regarded as the third generation of artificial neural networks [3], have attracted broad interest in bioinspired computing and neuromorphic systems research owing to their inherent sensitivity to temporal information [4,5]. In parallel, adaptive learning architectures developed from an engineering perspective have increasingly addressed temporal feature learning and unsupervised representation learning. A notable recent example is the Type-3 Adaptive Neuro-Fuzzy Inference System (T3-ANFIS) proposed by Mohammadzadeh et al. [6], who introduces a noniterative closed-form learning scheme that achieves efficient online parameter adaptation without iterative weight updates, underscoring the broad relevance of adaptive weight regulation across both biological and engineering paradigms. Multilayer feedforward architectures represent a fundamental organizational principle of cortical information processing, through which neural representations can progressively evolve from low-level sensory features to higher-level abstract representations along the hierarchical direction. Multilayer feedforward SNNs based on spike-timing-dependent plasticity (STDP) have demonstrated computational properties consistent with this hierarchical organizational principle in unsupervised feature learning [7,8], thereby providing an important bioinspired computational framework for investigating cortical hierarchical learning mechanisms.

STDP is considered one of the key mechanisms underlying long-term synaptic modification in biological neural systems [9]. In the canonical STDP rule, a synapse undergoes long-term potentiation (LTP) when a presynaptic spike precedes a postsynaptic spike, and long-term depression (LTD) in the reverse order. This Hebbian competitive learning mechanism, dependent on the relative timing of spikes [10], can selectively strengthen synaptic pathways that are highly correlated with input activity and supports the self-organization of stimulus-selective structures [11]. These functional properties have been experimentally supported in multiple brain regions including the cortex, hippocampus, and auditory system [9,12], and have been applied in multilayer SNNs to drive progressive abstraction of inter-layer features [7,8]. However, systematic quantitative analyses of how STDP learning dynamics evolve with layer depth in multilayer feedforward networks, and how inter-layer information is maintained during synaptic plasticity-driven reorganization, remain insufficient [8].

Real cortical networks are not composed purely of excitatory neurons, but rather form local circuits jointly by excitatory (E) and inhibitory (I) neurons in an approximate ratio of 4:1 [13,14]. Among these, regular spiking (RS) pyramidal neurons and fast spiking (FS) inhibitory interneurons represent two canonical cell types in cortical local circuits [14]. RS neurons typically exhibit firing rate adaptation, manifested as a gradual decrease in firing rate under sustained input; FS neurons are characterized by rapid, low-adaptation firing and can regulate the spike timing of RS neurons through fast lateral inhibition [14,15]. Excitatory–inhibitory (E-I) dynamic balance is considered an important basis for maintaining stable activity and achieving efficient neural coding in cortical networks [16,17], and its dysregulation has been implicated in the pathological mechanisms of various neuropsychiatric disorders including epilepsy and autism spectrum disorder [2].

Recent studies have demonstrated that inhibitory synapses also exhibit activity-dependent plasticity in the form of inhibitory spike-timing-dependent plasticity (iSTDP). iSTDP can participate in maintaining the E-I dynamic balance of networks by adaptively adjusting FS→RS inhibitory strength [18]. Prior work has shown that iSTDP can expand the parameter range over which excitatory STDP (eSTDP) operates stably [19], and promotes autonomous balance of excitatory–inhibitory activity in sensory pathways and memory networks [18]. Furthermore, appropriately enhanced inhibition may strengthen excitatory synaptic competition regulated by eSTDP [19]; when activity-dependent plasticity alone is insufficient to maintain stability, homeostatic plasticity mechanisms such as synaptic scaling can provide additional regulation [20]. Although unsupervised competitive learning frameworks based on the cooperative action of STDP and lateral inhibition have been shown to promote the emergence of neuronal selectivity [21,22], existing studies have largely focused on shallow networks or local circuits, and systematic investigation of the cooperative effects of eSTDP and iSTDP and their cross-layer propagation in multilayer feedforward networks remains limited.

At the level of neural coding, sparse coding is considered an important strategy by which the cortex represents natural stimuli [23,24]. The efficient coding hypothesis proposed by Barlow suggests that the nervous system can improve representational efficiency by reducing signal redundancy [25]; Olshausen and Field further demonstrated that learning a coding dictionary for natural images under sparsity constraints yields localized, oriented, bandpass features resembling the receptive fields of V1 simple cells [23,24]. Sparse coding is typically quantified by two classes of metrics, Lifetime Sparseness ($S_{L}$) and Population Sparseness ($S_{P}$) [26]: the former reflects the selectivity of individual neurons across different stimuli, while the latter reflects the sparseness of population responses to a given stimulus. However, in multilayer SNNs containing E-I local circuits, the relative contributions of RS firing rate adaptation and FS lateral inhibition to the formation of sparse coding, as well as their cooperative relationship, have not been clearly separated or quantified.

At the level of temporal pattern recognition, signal detection theory provides a standard framework for quantifying pattern discrimination ability [27]. The selectivity index $d^{\prime }$ and the area under the receiver operating characteristic (ROC) curve (AUC) are commonly used to evaluate the ability of a network to discriminate target patterns from distractor patterns. Information-theoretic methods, particularly mutual information I(S;R) [28], can characterize the encoding quality of neural population responses with respect to stimulus information and evaluate information transmission efficiency in multilayer networks. Prior work has shown that STDP is closely related to cortical temporal coding [29], and inhibitory circuits can enhance selective learning of specific temporal structures through temporal gating [30]. Nevertheless, how intra-layer FS connectivity affects temporal pattern learning capacity in multilayer feedforward networks, and whether it contributes to maintaining deep-layer information transmission efficiency, remain to be quantitatively clarified.

In summary, existing research still has the following limitations: (1) the regulatory role of intra-layer RS-FS circuits on cross-layer eSTDP learning dynamics remains unclear; (2) the influence of iSTDP on the stable learning region in the multidimensional E-I parameter space and its dynamic process lack systematic quantification; (3) the relative contributions of RS firing rate adaptation and FS lateral inhibition to sparse coding formation in multilayer networks have not been sufficiently separated; and (4) the effects of intra-layer FS connectivity on temporal pattern selective learning and deep-layer information transmission efficiency lack quantitative analysis.

To address these issues, we constructed an eight-layer feedforward RS-FS spiking neural network incorporating intra-layer E-I connectivity, employed the Izhikevich model [31] to describe the heterogeneous firing characteristics of RS and FS neurons, and systematically investigated the multilayer learning dynamics under the cooperative action of eSTDP and iSTDP. The main contributions of this paper are as follows:

1. We analyzed the influence of intra-layer FS lateral inhibition on cross-layer E→E weight evolution, demonstrating that it can alleviate the LTD bias induced by RS firing rate adaptation through the winner-take-all (WTA) competition mechanism and promote heterogeneous differentiation of E→E weights while maintaining stable mean synaptic strength. Whereas Vogels et al. [18] established that iSTDP maintains E-I balance in single-layer sensory and memory networks, the present work reveals an additional functional role of intra-layer FS inhibition in multilayer feedforward architectures: beyond stabilizing network activity, it actively drives competitive Hebbian differentiation of E→E weights across layers, effectively transforming inhibitory circuits from passive regulators into active promoters of synaptic diversity.
2. Through a 12×12 parameter grid scan, we evaluated the effect of iSTDP on the stable learning region in the E-I parameter space and revealed a sustained cooperative convergence pattern of eSTDP and iSTDP during training. Whereas Luz and Shamir [19] derived theoretical bounds on the stabilizing effect of Hebbian inhibitory plasticity in a single-layer setting, the present work provides systematic numerical quantification of this effect in a multilayer feedforward architecture and further reveals the cooperative convergence dynamics of eSTDP and iSTDP—specifically, the monotonic evolution of the E/I weight ratio toward a new equilibrium—a phenomenon that cannot be observed within a single-layer theoretical framework.
3. Through controlled experiments, we separated the contributions of RS firing rate adaptation and FS lateral inhibition to sparse coding formation, and found that sparse coding in the complete model evolves along the feedforward direction following a pattern of lower sparseness at the input layer, rapid increase at the second layer, and maintenance of a plateau in deeper layers.
4. Combining the signal detection metrics $d^{\prime }$ and AUC [27] with mutual information I(S;R) [28], we quantitatively evaluated the effects of intra-layer FS connectivity on temporal pattern selective learning and deep-layer information transmission efficiency. Building on the assembly formation framework of Litwin-Kumar and Doiron [30], we further demonstrate that fixed (non-plastic) intra-layer FS connections are sufficient to substantially improve temporal discrimination, and validate the robustness of this advantage across inter-group intervals and pattern types through systematic sensitivity and generalization tests.

These findings contribute to a deeper computational understanding of cortical E-I cooperative plasticity mechanisms, and provide bioinspired references for the design of neuromorphic computing systems with adaptive E-I balance capability.

## 2. Materials and Methods

### 2.1. Network Architecture

**Figure 1:**
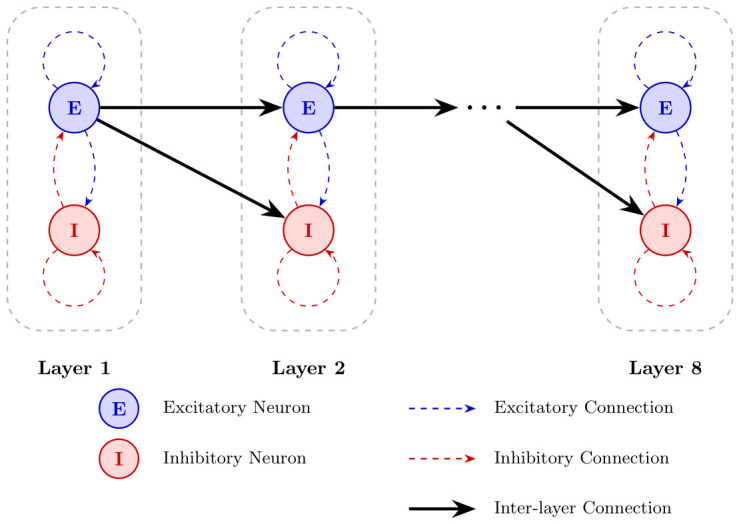
Schematic diagram of the eight-layer feedforward excitatory–inhibitory spiking neural network. Each layer comprises excitatory (E, RS) and inhibitory (I, FS) neurons. Blue dashed arrows indicate intra-layer excitatory connections; red dashed arrows indicate intra-layer inhibitory connections; black solid arrows indicate inter-layer excitatory (E→E) connections subject to eSTDP.

The choice of eight processing layers is motivated by the hierarchical organization of the primate visual cortex, which comprises approximately six to eight successive cortical processing stages from primary visual cortex (V1) through inferotemporal cortex (IT) [32]; this depth affords sufficient hierarchical stages to examine cross-layer learning dynamics while remaining computationally tractable. We constructed an eight-layer feedforward spiking neural network. Each layer contained 200 neurons, of which 160 were excitatory regular spiking (RS) neurons and 40 were inhibitory fast spiking (FS) neurons, yielding an excitatory-to-inhibitory neuron ratio of 4:1. Inter-layer connections existed exclusively between RS neurons in adjacent layers, i.e., E→E connections from RS neurons in layer *l* to RS neurons in layer l+1, with a connection probability of 0.50. Inter-layer E→E synaptic weights were initialized with a mean value plus 5% Gaussian noise; the initial mean was set to 0.28 for Section 3.1 and Section 3.2, and to 0.32 for Section 3.3 and Section 3.4. Within each layer, four types of intra-layer connections were established: RS→RS, RS→FS, FS→RS, and FS→FS, with corresponding connection probabilities of 0.15, 0.30, 0.30, and 0.15, respectively. Self-connections were excluded from all intra-layer connectivity. Intra-layer connections were used to simulate lateral excitation and lateral inhibition in cortical local E-I circuits. The overall network architecture is illustrated in Figure 1.

### 2.2. Neuron Model

All neurons were described using the Izhikevich model [31], in which the membrane potential *v* and recovery variable *u* evolve according to:

(1) $$\frac{dv}{dt}=0.04v^{2}+5v+140-u-I_{\mathrm{syn}}$$

(2) $$\frac{du}{dt}=a\left(bv-u\right)$$

When the membrane potential satisfies v≥30 mV, the neuron generates an action potential and is reset to v←c, u←u+d, where $I_{\mathrm{syn}}$ denotes the synaptic input current and *a*, *b*, *c*, *d* are model parameters governing the neuronal firing dynamics.

To simulate the firing rate adaptation of RS neurons, individual heterogeneity was introduced into the RS neuron parameters: a=0.02, b=0.20, $c=-65+15r^{2}$, $d=8-6r^{2}$, where r∼U(0,1). To simulate the rapid, low-adaptation firing characteristics of FS neurons, the FS neuron parameters were set to: a=0.02+0.08r, b=0.25−0.05r, c=−65, d=2. All simulations were performed with a fixed time step of dt=0.1 ms.

### 2.3. Synapse Model

Synaptic currents were described using the alpha-function model. The synaptic conductance g(t) evoked by a single synaptic event is given by:

(3) $$g\left(t\right)=\frac{w\cdot t}{\tau }e^{-t/\tau }$$

where *w* is the synaptic weight, τ is the synaptic time constant, and *t* is the time elapsed since the presynaptic spike. The postsynaptic current is jointly determined by excitatory and inhibitory conductances:

(4) $$I_{\mathrm{syn}}\left(t\right)=g_{\mathrm{exc}}\left(t\right)\cdot \left(v-V_{\mathrm{exc}}\right)+g_{\mathrm{inh}}\left(t\right)\cdot \left(v-V_{\mathrm{inh}}\right)$$

where $g_{\mathrm{exc}}\left(t\right)$ and $g_{\mathrm{inh}}\left(t\right)$ denote the excitatory and inhibitory synaptic conductances, respectively, and $V_{\mathrm{exc}}$ and $V_{\mathrm{inh}}$ are the corresponding reversal potentials. The excitatory synaptic time constant was set to $\tau_{\mathrm{exc}}=0.3$ ms, the inhibitory synaptic time constant to $\tau_{\mathrm{inh}}=0.6$ ms, the excitatory reversal potential to $V_{\mathrm{exc}}=0$ mV, and the inhibitory reversal potential to $V_{\mathrm{inh}}=-80$ mV.

Except for the parameter scan in Section 2.5, intra-layer excitatory synaptic weights were fixed at $g_{\mathrm{intra},\mathrm{exc}}=0.12$ and intra-layer inhibitory synaptic weights at $g_{\mathrm{intra},\mathrm{inh}}=0.096$. Neurons in the first layer additionally received Poisson spike input with strength $g_{\mathrm{Poisson}}=2.0$.

### 2.4. Synaptic Plasticity Rules

Two types of spike-timing-dependent plasticity rules were implemented: excitatory STDP (eSTDP) and inhibitory STDP (iSTDP).

#### 2.4.1. Excitatory STDP

eSTDP acted exclusively on E→E synapses between RS neurons in adjacent layers. A standard asymmetric exponential time window was adopted:

(5) $$\Delta w=\left\{\begin{array}{cc} A_{+}\cdot x_{\mathrm{pre}} & (\mathrm{LTP},\;\mathrm{upon}\;\mathrm{postsynaptic}\;\mathrm{RS}\;\mathrm{spike})\\ -A_{-}\cdot x_{\mathrm{post}} & (\mathrm{LTD},\;\mathrm{upon}\;\mathrm{presynaptic}\;\mathrm{RS}\;\mathrm{spike}) \end{array}\right.$$

where $x_{\mathrm{pre}}$ and $x_{\mathrm{post}}$ denote the presynaptic and postsynaptic spike traces, both decaying exponentially with time constants $\tau_{+}=\tau_{-}=20$ ms. The eSTDP learning rate parameters were set to $A_{+}=0.004$ and $A_{-}=0.008$, with $A_{-}>A_{+}$ so that LTD slightly dominates LTP, preventing unbounded growth of excitatory weights during training. Hard boundary clipping was applied to E→E weights: [0,0.45] in Section 3.1, and [0,0.50] in Section 3.3 and Section 3.4.

#### 2.4.2. Inhibitory STDP

iSTDP acted on intra-layer FS→RS inhibitory synapses. A symmetric plasticity rule was adopted:

(6) $$\Delta w_{\mathrm{inh}}=\left\{\begin{array}{cc} B_{+}\cdot x_{\mathrm{pre}} & (\mathrm{LTP},\;\mathrm{upon}\;\mathrm{postsynaptic}\;\mathrm{RS}\;\mathrm{spike})\\ -B_{-}\cdot x_{\mathrm{post}} & (\mathrm{LTD},\;\mathrm{upon}\;\mathrm{presynaptic}\;\mathrm{FS}\;\mathrm{spike}) \end{array}\right.$$

where $x_{\mathrm{pre}}$ and $x_{\mathrm{post}}$ denote the spike traces of the FS and RS neurons, respectively. The iSTDP time constants were set to $\tau_{+}=\tau_{-}=20$ ms, and the learning rate parameters to $B_{+}=B_{-}=0.002$. FS→RS inhibitory weights were constrained to the range [0,0.30].

### 2.5. Experimental Design and Control Conditions

To systematically analyze the effects of intra-layer E-I circuits, eSTDP, and iSTDP on multilayer learning dynamics, multiple controlled experiments were designed.

First, to examine the influence of intra-layer connectivity on eSTDP learning dynamics, we compared the evolution of E→E cross-layer weights under two conditions: a network without intra-layer connections (Condition A) and a network with intra-layer RS lateral excitation and FS lateral inhibition (Condition B).

Second, to evaluate the regulatory effect of iSTDP on the stable region of the E-I parameter space, a 12×12 parameter grid scan was performed over intra-layer excitatory strength $g_{\mathrm{exc}}\in \left[0.03,0.45\right]$ and FS inhibitory strength $g_{\mathrm{inh}}\in \left[0.01,0.35\right]$, comparing network stability under eSTDP-only and eSTDP+iSTDP conditions.

Third, to separate the contributions of RS firing rate adaptation and FS lateral inhibition to sparse coding formation, three conditions were defined: RS adaptation retained with intra-layer FS connections disabled (Condition A); RS adaptation removed with intra-layer FS connections retained (Condition B); and the complete model retaining both mechanisms (Condition C).

Finally, to evaluate the network’s capacity to learn specific temporal input patterns, a target temporal pattern and an equal-intensity random pattern were designed as test inputs, and the network’s response differences and layer-wise information transmission efficiency were assessed using the selectivity index, ROC/AUC, and mutual information.

### 2.6. Analysis Metrics

#### 2.6.1. Synaptic Weight Statistics

During training, the mean, standard deviation, and variance of all active E→E synaptic weights were computed. An active synapse was defined as one with weight greater than zero. The corresponding statistics are denoted $\bar{w}$, $\sigma_{w}$, and Var(w), where $\bar{w}$ characterizes the overall synaptic strength level, and $\sigma_{w}$ and Var(w) quantify the degree of synaptic weight differentiation.

The E/I weight ratio was defined as the ratio of the mean E→E weight to the mean FS→RS weight in the current layer:

(7) $$E/I\;\mathrm{ratio}=\frac{{\bar{w}}_{E\to E}}{{\bar{w}}_{FS\to RS}}$$

This metric describes the relative change between excitatory and inhibitory synaptic strengths during training.

#### 2.6.2. Lifetime Sparseness

Lifetime Sparseness ($S_{L}$) quantifies the selectivity of individual neurons across different stimuli and is defined as:

(8) $$S_{L}=\frac{1-{\left(\sum_{s=1}^{n}r_{s}/n\right)}^{2}/\left(\sum_{s=1}^{n}r_{s}^{2}/n\right)}{1-1/n}$$

where $r_{s}$ is the mean firing rate of the neuron in response to the *s*-th stimulus, and *n* is the total number of stimuli (n=30 in this study). $S_{L}$ ranges from 0 to 1: $S_{L}\to 1$ indicates that the neuron responds strongly to only a few stimuli (high selectivity), while $S_{L}\to 0$ indicates uniform responses across all stimuli (no selectivity).

#### 2.6.3. Population Sparseness

Population Sparseness ($S_{P}$) quantifies the proportion of neurons in the population that participate in the response to a given stimulus. It is defined in the same form as $S_{L}$, but computed along the neuron dimension rather than the stimulus dimension:

(9) $$S_{P}=\frac{1-{\left(\sum_{i=1}^{M}r_{i}/M\right)}^{2}/\left(\sum_{i=1}^{M}r_{i}^{2}/M\right)}{1-1/M}$$

where $r_{i}$ is the mean firing rate of the *i*-th neuron in response to the current stimulus, and *M* is the total number of neurons in the population. $S_{P}\to 1$ indicates that only a small number of neurons respond (sparse activation), while $S_{P}\to 0$ indicates uniform responses across the entire population (dense activation).

#### 2.6.4. Selectivity Index $d^{\prime }$ and ROC/AUC

The selectivity index $d^{\prime }$, based on signal detection theory [27], quantifies the ability of the network to discriminate between a target temporal pattern and an equal-intensity random pattern:

(10) $$d^{\prime }=\frac{\mu_{\mathrm{target}}-\mu_{\mathrm{random}}}{\sqrt{(\sigma_{\mathrm{target}}^{2}+\sigma_{\mathrm{random}}^{2})/2}}$$

where $\mu_{\mathrm{target}}$ and $\mu_{\mathrm{random}}$ are the mean RS population firing rates in response to the target and random patterns, respectively, and $\sigma_{\mathrm{target}}$, $\sigma_{\mathrm{random}}$ are the corresponding standard deviations. $d^{\prime }=0$ indicates no discriminability between the two pattern types, while $d^{\prime }\geq 1$ is commonly used as a reference threshold for strong pattern discrimination. The ROC curve traces the trade-off between the true positive rate (TPR) and the false positive rate (FPR) across decision thresholds, and the area under the curve (AUC) reflects the overall discriminative performance of the classifier: AUC=0.5 corresponds to chance-level performance and AUC=1.0 to perfect classification.

#### 2.6.5. Mutual Information I(S;R)

Mutual information quantifies the statistical dependence between the RS population firing rate at each layer and the input stimulus frequency, and is defined as [28]:

(11) $$I\left(S;R\right)=\sum_{s}\sum_{r}p\left(s,r\right)\log_{2}\frac{p(s,r)}{p(s)\;p(r)}$$

where *S* denotes the stimulus category (30 Poisson frequencies) and *R* denotes the discretized bins of the RS population mean firing rate. A histogram estimation method was used in this study, with firing rate responses uniformly divided into 10 bins. I(S;R) was computed layer-wise (in bits) to quantify the encoding quality of each layer with respect to stimulus frequency information. A higher I(S;R) value indicates that the population response at that layer provides greater discriminability across different stimuli.

### 2.7. Simulation Procedure

Prior to each simulation, the network underwent a 50 ms warm-up phase to reduce the influence of initial transient activity on the training process. This was followed by the STDP training phase, with a maximum training duration of 8000 ms and a simulation time step of dt=0.1 ms.

During training, eSTDP and iSTDP were updated synchronously according to their respective target synapse types. A snapshot of synaptic weights in each layer was recorded every 1000 ms to analyze the temporal evolution of weight mean, variance, and E/I weight ratio.

After training, all synaptic weights were fixed and test stimuli were injected into the network. The RS neuron population firing responses at each layer were recorded during the test phase for computing sparse coding metrics, the selectivity index, ROC/AUC, and mutual information. To ensure comparability across conditions, all controlled experiments used identical network initialization and random seed settings.

## 3. Results

### 3.1. Effect of Intra-Layer Connectivity on eSTDP-Mediated E→E Weight Differentiation

In a purely feedforward network, the learning efficacy of eSTDP depends on the precise spike timing relationship between presynaptic and postsynaptic neurons. The intrinsic firing rate adaptation of RS neurons may cause relative delays in postsynaptic firing, making it more likely for some pre-post spike pairs to fall within the LTD time window, thereby limiting effective differentiation of E→E synaptic weights. Intra-layer fast lateral inhibition mediated by FS neurons can regulate the spike timing of the RS population, but its specific influence on multilayer feedforward eSTDP learning dynamics requires further analysis. To this end, we compared the evolution of E→E cross-layer weights under two network configurations: a network without intra-layer connections (Condition A) and a network with intra-layer RS lateral excitation and FS lateral inhibition (Condition B).

**Figure 2:**
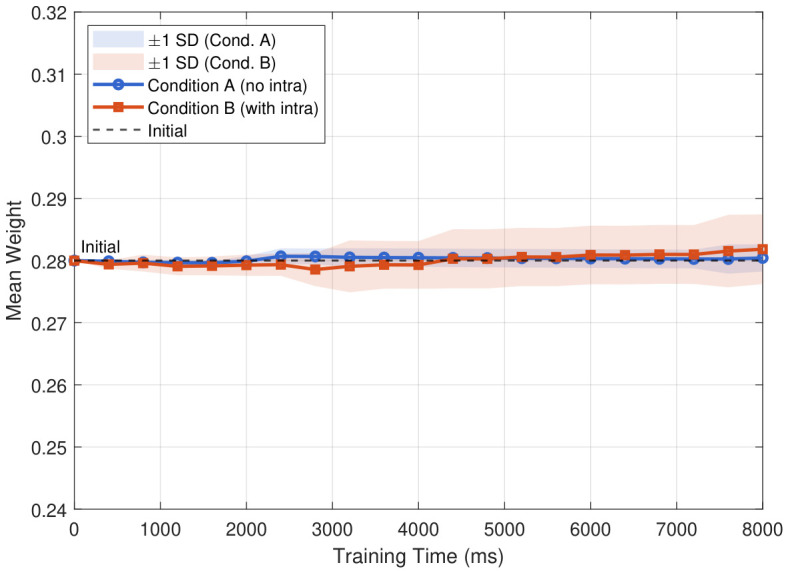
Evolution of mean E→E cross-layer weights with and without intra-layer connections under eSTDP. Shaded bands indicate ±1 standard deviation across five independent runs with different random seeds. The dashed line denotes the initial weight value.

As shown in Figure 2, after 8000 ms of training, the mean E→E weights under both conditions remained close to the initial value (approximately 0.28); at t=8000 ms, the mean weights of Condition A and Condition B were 0.2804±0.0022 and 0.2818±0.0056, respectively, showing no significant difference (paired *t*-test: p=0.469; Mann–Whitney *U* test: p=0.841). This indicates that neither network configuration exhibited pronounced weight explosion or learning extinction under the current STDP parameter settings. Although the mean weight trajectories were similar across conditions, the degree of weight dispersion differed markedly. The standard deviation range of Condition B began to expand after approximately 1000 ms of training, whereas that of Condition A remained narrow throughout the entire training process. This indicates that intra-layer connectivity did not substantially alter the mean E→E synaptic strength, but primarily affected the weight distribution within the synaptic population, inducing more pronounced heterogeneous differentiation while keeping the overall mean relatively stable.

**Figure 3:**
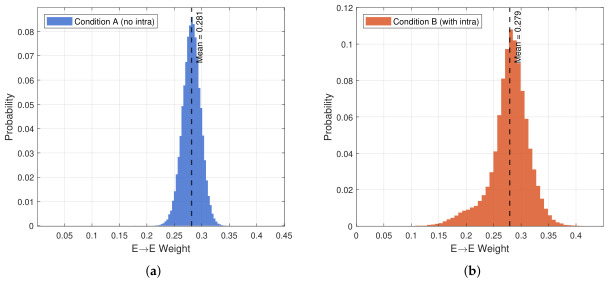
Steady-state E→E weight distributions after training with and without intra-layer connections under eSTDP. (**a**) Condition A (without intra-layer connections). (**b**) Condition B (with intra-layer connections). Dashed vertical lines indicate the mean weight value for each condition.

Figure 3a,b further illustrates the difference in E→E weight distributions after training. Under Condition A (Figure 3a), the weight distribution exhibited a concentrated unimodal shape, largely confined to the narrow range of 0.27–0.30, indicating that E→E synapses remained highly homogeneous in the absence of intra-layer competition. By contrast, the weight distribution under Condition B (Figure 3b) was markedly broader, spanning approximately 0.15–0.40, with a right-skewed tail in the high-weight region. This result demonstrates that the intra-layer RS-FS circuit can promote differentiation of synaptic weights from an initially homogeneous state toward a heterogeneous one.

This weight differentiation is likely related to the WTA competition mediated by FS lateral inhibition. Under intra-layer inhibition, RS neurons that fire earlier or more strongly are more likely to retain their firing advantage, causing their associated feedforward synapses to receive more LTP opportunities; neurons with weaker or delayed responses are more readily suppressed, and their associated synapses are more likely to accumulate LTD. Consequently, intra-layer FS lateral inhibition enhances competitive differentiation among E→E synapses without substantially altering the mean weight.

**Figure 4:**
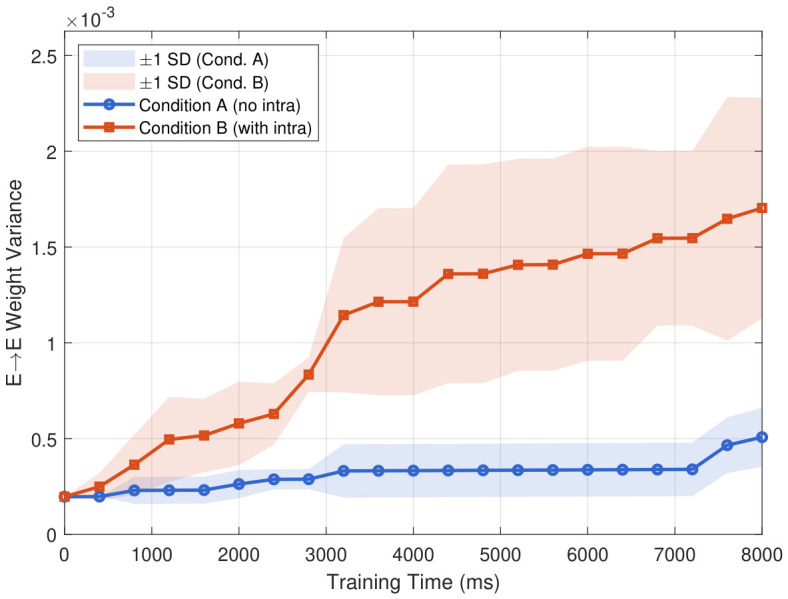
Evolution of E→E weight variance with and without intra-layer connections under eSTDP. Shaded bands indicate ±1 standard deviation across five independent runs with different random seeds.

Figure 4 further quantifies the weight differentiation process over time. The weight variance of Condition A remained low throughout training, increasing gradually from approximately $2.5\times 10^{-4}$ to $5.07\times 10^{-4}$ by 8000 ms, indicating that feedforward eSTDP alone produces only limited synaptic differentiation in the absence of intra-layer competition. By contrast, the weight variance of Condition B increased continuously from the onset of training, reaching $1.70\times 10^{-3}$ at 8000 ms—a 3.36-fold increase over Condition A (paired *t*-test: p=0.012; Mann–Whitney *U* test: p=0.008). This result demonstrates that intra-layer connectivity can significantly enhance eSTDP-induced E→E weight differentiation.

The continuous rise of weight variance in Condition B throughout training, without reaching a stable plateau, suggests that with fixed FS inhibitory weights, the intra-layer inhibitory strength becomes progressively insufficient to counterbalance the accumulating excitatory weight divergence. Therefore, relying solely on fixed inhibitory connections may be inadequate for long-term stabilization of multilayer eSTDP learning, which motivates the subsequent introduction of iSTDP for adaptive inhibitory regulation.

### 3.2. E-I Parameter Space and Cooperative Regulation by iSTDP

To examine the effects of E-I parameter configuration and iSTDP on network learning stability, a 12×12 log-uniformly spaced parameter grid was used to scan the two-dimensional space defined by intra-layer excitatory connection strength $g_{\mathrm{exc}}\in \left[0.03,0.45\right]$ and FS inhibitory connection strength $g_{\mathrm{inh}}\in \left[0.01,0.35\right]$. E→E weight variance and RS population mean firing rate were adopted as two metrics for evaluating network steady state, and the spatial distributions under eSTDP-only and eSTDP+iSTDP conditions were compared.

**Figure 5:**
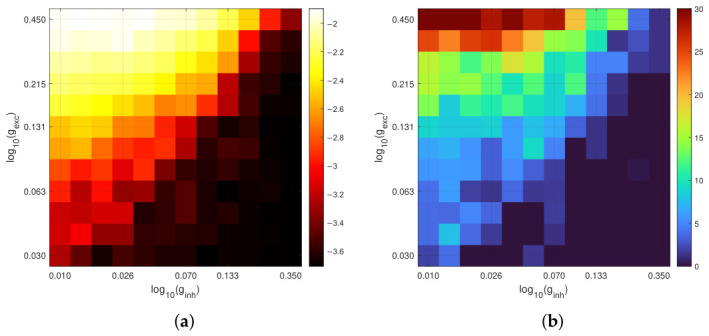
Stability distribution in the E-I parameter space under the eSTDP-only condition. The horizontal axis represents intra-layer inhibitory strength $\log_{10}\left(g_{\mathrm{inh}}\right)$ and the vertical axis represents intra-layer excitatory strength $\log_{10}\left(g_{\mathrm{exc}}\right)$. (**a**) E→E synaptic weight variance (log scale). (**b**) Mean RS population firing rate (Hz, capped at 30 Hz). Each grid cell represents the mean of three independent simulation runs.

Figure 5a,b shows the network stability distribution in the E-I parameter space under the eSTDP-only condition. The weight variance heatmap (Figure 5a) and RS firing rate heatmap (Figure 5b) jointly indicate that network state is sensitive to the combination of intra-layer excitatory and inhibitory strengths. In the high-$g_{\mathrm{exc}}$, low-$g_{\mathrm{inh}}$ region, excitatory drive is relatively strong, RS population firing rates can exceed 30 Hz, and E→E weight variance is at a high level, suggesting that the network may enter a state dominated by hyperexcitability and weight potentiation. In the low-$g_{\mathrm{exc}}$, high-$g_{\mathrm{inh}}$ region, strong FS inhibition suppresses RS population firing, leading to insufficient effective pre-post spike pairing and consequent suppression of eSTDP learning activity. The diagonal region between these two extremes exhibited moderate RS firing rates and weight variance, and can be regarded as a relatively stable learning zone. Overall, in the absence of inhibitory plasticity regulation, the stable learning zone is largely confined to a narrow range of E-I parameter combinations, indicating that multilayer eSTDP learning with fixed inhibition is sensitive to parameter configuration.

**Figure 6:**
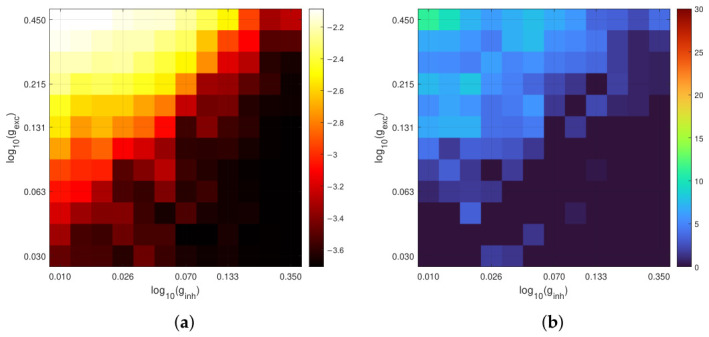
Stability distribution in the E-I parameter space under the eSTDP+iSTDP condition. Axes are identical to Figure 5. (**a**) E→E synaptic weight variance (log scale). (**b**) Mean RS population firing rate (Hz, capped at 30 Hz). Each grid cell represents the mean of three independent simulation runs.

Figure 6a,b shows the stability distribution in the same parameter space after introducing iSTDP. Compared with the eSTDP-only condition, the addition of iSTDP produced notable changes in the spatial distributions of both RS firing rate (Figure 6b) and E→E weight variance (Figure 6a). First, in the high-$g_{\mathrm{exc}}$, low-$g_{\mathrm{inh}}$ region, the originally elevated RS firing rates were partially suppressed, suggesting that iSTDP can form negative feedback regulation of excessively strong excitatory activity by enhancing FS→RS inhibitory weights. Second, the region of effective firing extended toward lower $g_{\mathrm{exc}}$ values; in some low-excitation parameter regions that were near-silent under eSTDP only, the network was able to maintain low but non-zero RS firing activity after the introduction of iSTDP. Concurrently, the high-variance region in the weight variance heatmap contracted, consistent with the trend in firing rate distribution.

It should be noted that iSTDP provides limited improvement in the learning-extinction region under strong inhibition and low excitation. When RS population firing is excessively suppressed, iSTDP lacks sufficient postsynaptic spike signals to trigger weight updates and therefore cannot independently restore network activity. Overall, the introduction of iSTDP expanded the region of the E-I parameter space in which effective learning can be maintained and improved the network’s adaptability to moderate parameter mismatch.

**Figure 7:**
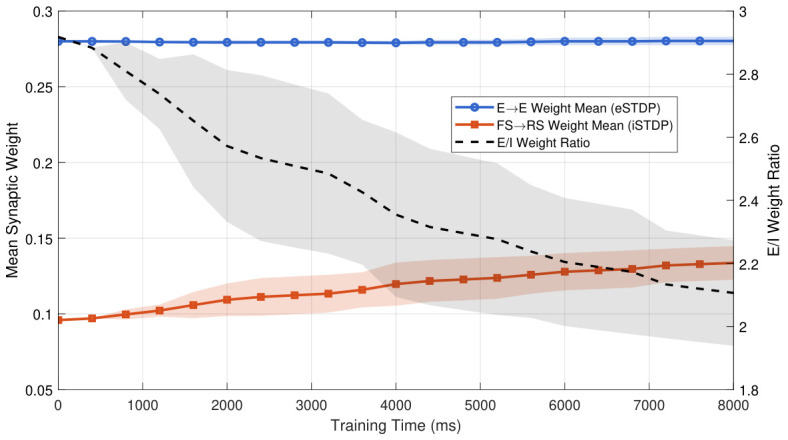
Evolution of mean E→E weight (eSTDP), mean FS→RS weight (iSTDP), and E/I weight ratio over 8000 ms of training under $g_{\mathrm{intra},\mathrm{exc}}=0.12$ and $g_{\mathrm{intra},\mathrm{inh}}=0.096$. Shaded bands indicate ±1 standard deviation across five independent runs with different random seeds. The left axis corresponds to synaptic weight values and the right axis to the E/I weight ratio.

To further analyze the temporal course of iSTDP regulation, Figure 7 records the trajectories of the mean E→E weight, mean FS→RS weight, and E/I weight ratio over 8000 ms of training. The mean E→E cross-layer weight regulated by eSTDP remained close to its initial value of approximately 0.28 throughout training (0.2802±0.0028 at t=8000 ms; paired *t*-test: p=0.863), indicating that the introduction of iSTDP did not substantially disrupt the overall regulation of excitatory weights by eSTDP under this parameter configuration.

By contrast, the mean FS→RS inhibitory weight regulated by iSTDP gradually increased during training, and the E/I weight ratio declined continuously from an initial value of approximately 3.0 to 2.107±0.167 at t=8000 ms. Across independent runs, this decline was approximately monotonic throughout the training period, with the FS→RS weight reaching 0.1337±0.0111 (paired *t*-test: p=0.002) and the E/I weight ratio declining significantly from its initial value (paired *t*-test: p<0.001). This sustained evolution indicates that iSTDP progressively adjusts inhibitory weights in response to network activity, transitioning the E/I weight relationship from its initial state toward a new relative equilibrium. Meanwhile, the mean E→E weight remained relatively stable, suggesting that eSTDP and iSTDP can achieve cooperative regulation under this parameter configuration without exhibiting marked mutual interference.

### 3.3. Emergence of Sparse Coding

To separate the relative contributions of RS firing rate adaptation and FS lateral inhibition to sparse coding formation, three control conditions were established: Condition A retained RS firing adaptation with intra-layer FS connections disabled; Condition B removed RS firing adaptation while retaining intra-layer FS connections; and Condition C was the complete model retaining both mechanisms. Following 8000 ms of STDP training under each condition, 30 Poisson input stimuli of varying intensities were used as test stimuli, with input frequencies distributed log-uniformly in the range of 50–500 Hz; note that these test frequencies differ from the fixed training frequency (180 Hz), constituting a cross-frequency generalization evaluation consistent with the standard $S_{L}$ computation protocol [26]. Lifetime Sparseness ($S_{L}$) and Population Sparseness ($S_{P}$) were then computed to evaluate single-neuron selectivity and population response sparseness, respectively.

**Figure 8:**
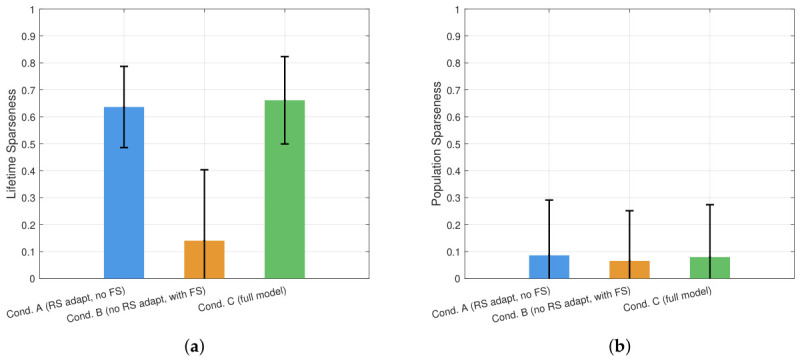
Comparison of contributions of RS adaptation and FS lateral inhibition to sparse coding under three control conditions: A (RS adaptation only), B (FS inhibition only), and C (complete model). Error bars denote standard deviations. (**a**) Lifetime Sparseness ($S_{L}$); error bars were computed across all RS neurons in all layers. (**b**) Population Sparseness ($S_{P}$); error bars were computed across all layers and all stimulus conditions.

As shown in Figure 8a, the three conditions exhibited marked differences in $S_{L}$. Under Condition A, $S_{L}=0.64$, indicating that with RS firing adaptation retained, individual neurons can form strong response selectivity across stimuli of different frequencies. This suggests that RS firing adaptation may be a primary factor promoting the formation of Lifetime Sparseness, likely because the adaptive decay of RS neuron responses to stimuli of varying intensities induces differentiation in response amplitude, thereby enhancing stimulus selectivity at the single-neuron level. By contrast, $S_{L}$ under Condition B was only 0.15, indicating that with RS firing adaptation removed, FS lateral inhibition alone is insufficient to produce high single-neuron sparseness. This result suggests that, under the present frequency-modulated stimulus conditions, the direct contribution of FS lateral inhibition to Lifetime Sparseness is relatively limited; its role is more likely to be expressed through WTA competition that amplifies the selectivity differences already established by RS adaptation. The complete model under Condition C yielded $S_{L}=0.67$, slightly higher than Condition A, indicating that the simultaneous presence of RS firing adaptation and FS lateral inhibition can further enhance single-neuron selectivity, although the primary contribution still originates from RS firing adaptation.

Regarding $S_{P}$ (Figure 8b), differences among the three conditions were small, with values ranging from approximately 0.05 to 0.09. This suggests that Population Sparseness is less sensitive to RS firing adaptation and FS lateral inhibition than Lifetime Sparseness, and may be more strongly influenced by the overall activity state and the degree of population synchrony. In addition, a parameter scan over FS inhibitory strength $g_{\mathrm{inh}}$ showed that as $g_{\mathrm{inh}}$ increased from 0.03 to 0.30, $S_{L}$ exhibited a moderate upward trend from 0.61 to 0.69, while $S_{P}$ remained essentially flat over this range. This indicates that, within the current parameter range, increasing FS inhibition can modestly improve single-neuron selectivity, but no clear optimal inhibitory strength was identified.

**Figure 9:**
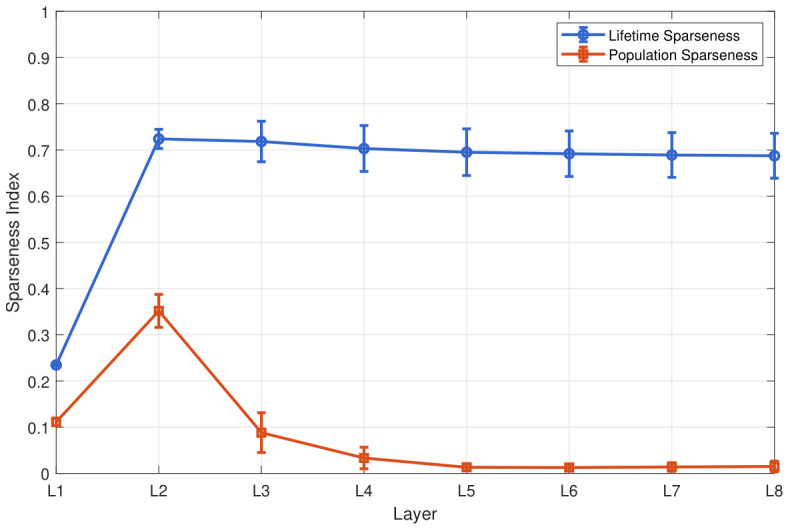
Layer-wise evolution of Lifetime Sparseness ($S_{L}$) and Population Sparseness ($S_{P}$) along the feedforward hierarchy in the complete model. Error bars indicate ±1 standard deviation across five independent runs with different random seeds.

Figure 9 shows the layer-wise changes in $S_{L}$ and $S_{P}$ along the feedforward hierarchy in the complete model. $S_{L}$ exhibits a low-high-plateau pattern across layers: $S_{L}$ at L1 is approximately 0.25, lower than subsequent layers; from L1 to L2, $S_{L}$ rises rapidly to approximately 0.73, a significant increase confirmed by paired *t*-test (p<0.001); thereafter, L2–L8 maintains a high-level plateau of approximately 0.74, with no significant difference between L2 and L8 (p=0.073). The relatively low $S_{L}$ at L1 is likely attributable to the fact that this layer receives direct Poisson input drive, causing most RS neurons to respond to changes in input frequency, resulting in relatively weak single-neuron selectivity. The rapid increase in $S_{L}$ from L1 to L2 suggests that inter-layer propagation may involve pronounced information filtering, with L1 neuron activity that is more strongly correlated with the input timing more readily driving L2 neuron firing. The maintenance of $S_{L}$ at a high plateau above L2 indicates that the selective response patterns formed after STDP training remain relatively stable along the feedforward direction.

The layer-wise trend of $S_{P}$ differs from that of $S_{L}$. $S_{P}$ at L1 is approximately 0.10, rises to a peak of approximately 0.35 at L2 (paired *t*-test, L1 vs. L2: p<0.001), and then gradually declines from L3 to L8, reaching approximately 0.02 in the deep layers (paired *t*-test, L2 vs. L8: p<0.001). The $S_{P}$ peak at L2 may reflect selective population activation during propagation from the input layer to the second layer, while the decline of $S_{P}$ in deeper layers suggests that deep-layer RS population responses may tend toward synchrony or homogenization, reducing the sparseness of population activation under specific stimuli. Overall, sparse coding in the complete model is predominantly formed during the L1-to-L2 propagation stage and is subsequently maintained in the form of high Lifetime Sparseness across deeper layers. The elevated error bars at L2 for both $S_{L}$ and $S_{P}$ reflect the stochastic nature of competitive weight differentiation at the first feedforward synaptic transformation. Because L1 neurons receive direct Poisson drive and respond primarily according to input frequency, their activity patterns are largely stimulus-determined and remain consistent across runs. The L1→L2 eSTDP synapses, however, constitute the first locus at which WTA competition drives heterogeneous weight differentiation: different random seeds produce different “winning” subsets of L2 neurons, resulting in substantial across-run variability in both the identity of selective neurons and the magnitude of their responses. Beyond L2, each successive layer receives an already sparse, selectivity-filtered input shaped by the preceding WTA process; the competitive dynamics consequently become progressively more constrained, and the across-run variability in $S_{L}$ and $S_{P}$ diminishes accordingly, as reflected in the narrowing error bars from L3 to L8.

### 3.4. Selective Temporal Pattern Learning and Layer-Wise Protection of Information Transmission

To examine whether the complete RS-FS network can learn specific input temporal patterns and form selective responses through STDP, 160 RS neurons in L1 were randomly divided into 8 groups of 20 neurons each. Groups fired sequentially at fixed intervals of 5 ms, forming a target temporal pattern lasting 35 ms. The training phase lasted 8000 ms, with the target pattern presented repeatedly every 200 ms; during the remaining intervals, random Poisson background input at 80 Hz was injected. Network temporal selectivity was then quantified using a multi-trial design (20 trials per condition), with $d^{\prime }$ and AUC [27] as the primary discrimination metrics.

**Figure 10:**
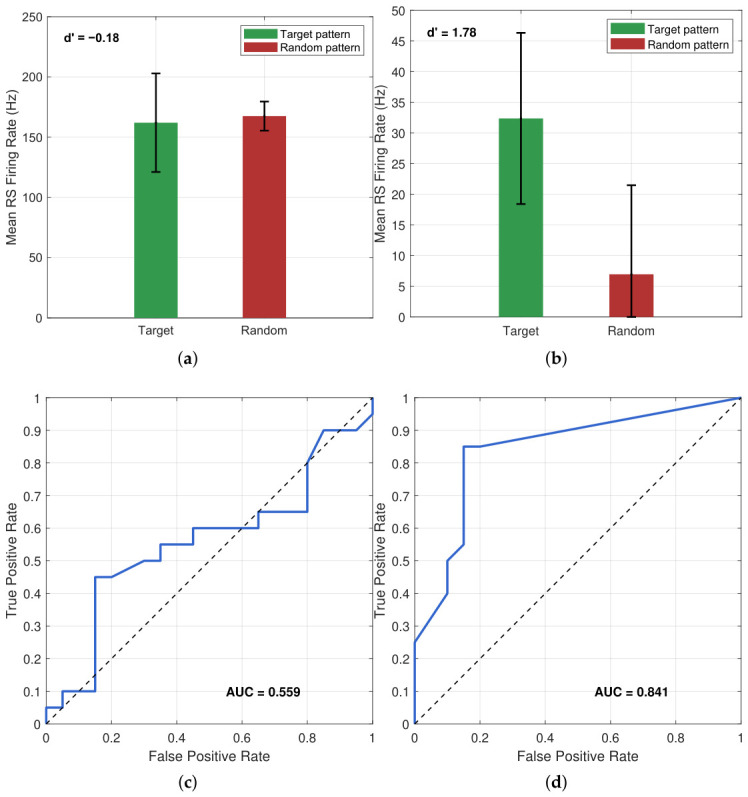
Selective responses of the network to the target temporal pattern before and after STDP training. Error bars indicate ±1 standard deviation across 20 test trials. (**a**) Mean RS population firing rates before training ($d^{\prime }=-0.18$). (**b**) Mean RS population firing rates after training ($d^{\prime }=1.78$). (**c**) ROC curve before training (AUC=0.559). (**d**) ROC curve after training (AUC=0.841). Dashed diagonal lines in (**c**,**d**) indicate chance-level performance.

**Figure 11:**
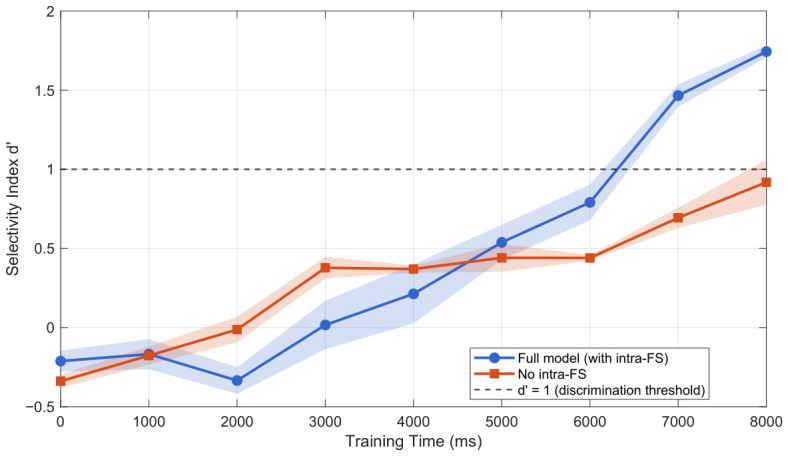
Evolution of the selectivity index $d^{\prime }$ over training time with and without intra-layer FS connections. Shaded bands indicate ±1 standard deviation across five independent runs with different random seeds. The dashed horizontal line indicates the $d^{\prime }=1$ discrimination reference threshold.

Figure 10 presents results from a single representative run to illustrate the qualitative change in network responses before and after STDP training; statistical reproducibility across five independent runs with different random seeds is subsequently quantified in Figure 11.

As shown in Figure 10a, prior to training the network showed no reliable discrimination between the target and random patterns, with mean RS population firing rates of approximately 163 Hz and 169 Hz, respectively, corresponding to $d^{\prime }=-0.18$ and AUC=0.559 (Figure 10c)—both near chance level. After 8000 ms of STDP training (Figure 10b), the network responses to the target and random patterns were approximately 32.5 Hz and 7.2 Hz, respectively; $d^{\prime }$ increased to 1.78 and AUC to 0.841 (Figure 10d), well exceeding the commonly used $d^{\prime }=1$ pattern discrimination reference threshold. The ROC curve shifted substantially toward the upper left after training (Figure 10d), indicating improved discriminability across all decision thresholds. Notably, the overall population firing rate decreased from approximately 163 Hz before training to approximately 32.5 Hz after training, indicating that STDP not only altered overall excitability but also sharpened the relative response difference between the target and random patterns.

Figure 11 shows the evolution of $d^{\prime }$ over training time with and without intra-layer FS connections. Both conditions started from negative $d^{\prime }$ values, reflecting the absence of reliable pattern discrimination prior to STDP-induced weight reorganization. Throughout training, $d^{\prime }$ increased monotonically on average under both conditions, with the complete model rising more steeply. At 8000 ms, $d^{\prime }$ in the complete model reached 1.743±0.038, exceeding the $d^{\prime }=1$ discrimination threshold; by contrast, the final $d^{\prime }$ under the no-FS condition was 0.918±0.141, remaining below threshold (Welch’s *t*-test: t=12.635, p<0.001). These results demonstrate that intra-layer FS connections substantially enhance selective learning of the target temporal pattern, with the final $d^{\prime }$ of the complete model approximately 1.90 times that of the no-FS condition.

To assess the sensitivity of the main results to the choice of group interval, we systematically varied the inter-group delay across five values (3, 5, 8, 10, and 15 ms) while keeping all other parameters identical to the main experiment. For each interval, both the complete model and the no-FS condition were independently trained five times, and pattern discrimination was evaluated using the same multi-trial $d^{\prime }$ protocol.

**Figure 12:**
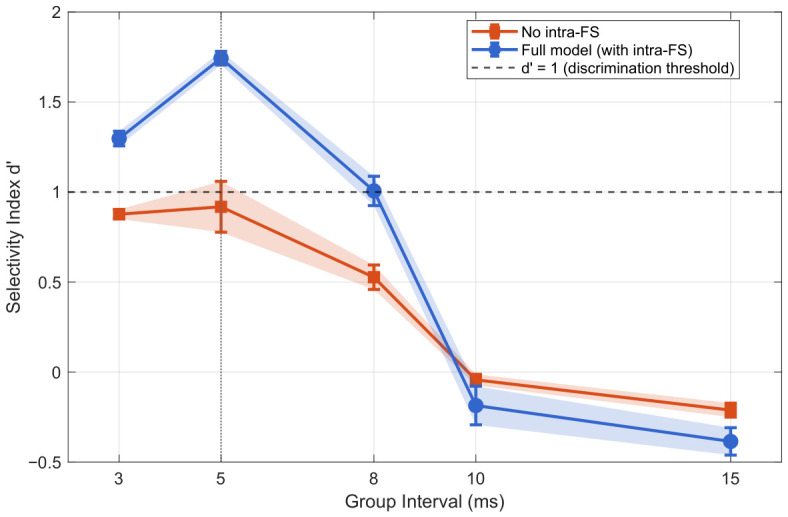
Sensitivity of $d^{\prime }$ to group interval (3, 5, 8, 10, and 15 ms) for the complete model and the no-FS condition. Error bars indicate ±1 standard deviation across five independent runs with different random seeds. The dashed horizontal line indicates the $d^{\prime }=1$ discrimination reference threshold, and the dotted vertical line marks the 5 ms interval used in the main experiment.

As shown in Figure 12, the complete model achieved $d^{\prime }>1$ at both 3 ms and 5 ms intervals, and approached the threshold at 8 ms, while the no-FS condition remained below $d^{\prime }=1$ across all tested intervals. At intervals of 3, 5, and 8 ms, the complete model significantly outperformed the no-FS condition (Welch’s *t*-test: all p<0.001); significant differences were also observed at 10 ms (p=0.039) and 15 ms (p=0.004), though both conditions yielded negative $d^{\prime }$ values at these longer intervals, indicating that reliable pattern discrimination broke down when the inter-group delay exceeded approximately 8 ms. These results demonstrate that the advantage of intra-layer FS connectivity in temporal pattern learning is robust across a range of biologically plausible inter-group intervals, and that the 5 ms interval used in the main experiment lies within the optimal operating range of the network.

To evaluate whether the network learned the temporal order structure of the input sequence rather than merely the identities of the activated neurons, we conducted a pattern generalization test using the trained weights from the complete model. Four pattern types were presented for 20 trials each: the original Target pattern (5 ms inter-group interval, fixed neuron grouping); a Jittered variant in which each group’s firing time was perturbed by a uniformly distributed offset of ±2 ms while preserving sequential order; a Shuffled variant in which the same neuron groups fired at the same time slots but in a randomly permuted order; and a Random pattern constructed from newly reassigned neuron groupings with the same fixed interval.

**Figure 13:**
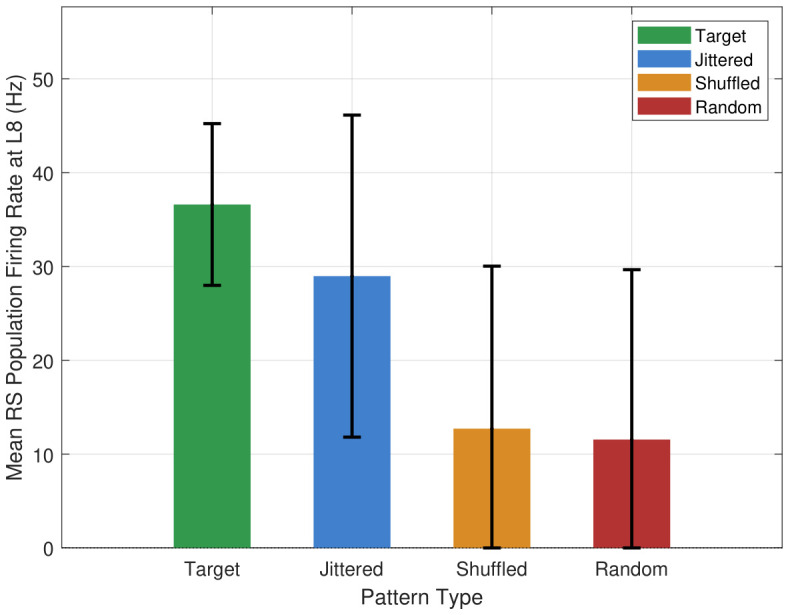
Mean RS population firing rate at L8 in response to four pattern types after STDP training. Error bars indicate +1 standard deviation (lower bound truncated at zero; n=20 trials per condition). Target: original target pattern; Jittered: temporally jittered version (±2 ms per group); Shuffled: order-shuffled version; Random: random neuron grouping with fixed interval.

As shown in Figure 13, the network responded most strongly to the Target pattern (approximately 37 Hz), followed by the Jittered pattern (approximately 29 Hz), while the Shuffled and Random patterns elicited substantially weaker responses (approximately 13 Hz and 12 Hz, respectively). The network responded significantly more strongly to the Target pattern than to the Shuffled and Random patterns (Welch’s *t*-test: both p<0.001), while no significant difference was found between Target and Jittered (p=0.086), or between Shuffled and Random (p=0.837). These results indicate that the network acquired selectivity primarily for the temporal order structure of the input sequence: responses were well preserved under small temporal jitter (±2 ms), but were substantially reduced when the sequential order was disrupted (Shuffled) or when the activated neuron identities were randomized (Random). The comparable responses to Shuffled and Random patterns further suggest that, after STDP training, the network’s selectivity depends critically on the specific firing sequence rather than on the identities of the active neurons per se.

**Figure 14:**
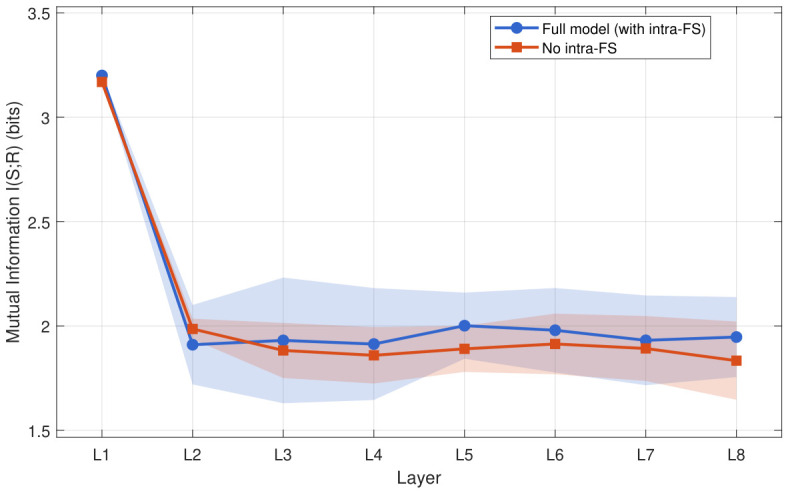
Layer-wise distribution of mutual information I(S;R) for RS populations after training, comparing the complete model and the condition without intra-layer FS connections. Error bars indicate ±1 standard deviation across five independent runs with different random seeds.

To evaluate the effect of intra-layer FS connections on information transmission, Figure 14 compares the layer-wise mutual information I(S;R) after training under the complete model and the no-FS condition. Both conditions showed a sharp drop from L1 (approximately 3.2 bits) to L2 (approximately 1.9 bits), with a significant between-condition difference at L1 (two-sample *t*-test: p<0.001), reflecting substantial information compression at the first feedforward synaptic transformation. From L2 onward, the complete model consistently maintained higher I(S;R) than the no-FS condition across all layers, with the largest difference near L3 (approximately 2.2 bits vs. 1.85 bits). Although individual layer-wise comparisons did not reach statistical significance (all p>0.05), likely due to limited statistical power with five independent runs, the consistent direction of the effect across L2–L8 points to a systematic tendency for intra-layer FS circuits to preserve more stimulus-related information during feedforward propagation—a conclusion further supported by the significant $d^{\prime }$ improvement observed in the complete model.

## 4. Discussion

### 4.1. Role of Intra-Layer FS Lateral Inhibition in eSTDP-Mediated Weight Differentiation

The present results demonstrate that intra-layer FS lateral inhibition promotes heterogeneous differentiation of E→E synaptic weights without substantially altering mean synaptic strength, increasing weight variance approximately 3.36-fold relative to the condition without intra-layer connectivity. This finding is consistent with the theoretical prediction that lateral inhibition facilitates competitive Hebbian learning by implementing a WTA mechanism [21], and extends this principle to the context of multilayer feedforward STDP learning. The WTA competition introduced by FS neurons preferentially allows RS neurons that fire earlier or more vigorously to accumulate LTP, while suppressing weaker or delayed neurons toward LTD, a process analogous to the competitive receptive field formation reported in shallow STDP networks [22]. Importantly, our results show that this competitive differentiation can be sustained across eight feedforward layers, suggesting that intra-layer E-I circuits may serve as a general mechanism for maintaining synaptic diversity in deep hierarchical architectures.

The continuous rise of E→E weight variance in Condition B throughout training, without reaching a stable plateau, further underscores the necessity of adaptive inhibitory regulation. As excitatory weights progressively differentiate, a fixed inhibitory tone becomes increasingly mismatched with the evolving excitatory landscape, potentially destabilizing the E-I balance. This observation motivates the use of iSTDP as a dynamic compensatory mechanism, consistent with the theoretical framework proposed by Vogels et al. [18], in which inhibitory plasticity continuously tracks excitatory activity to maintain network stability.

### 4.2. iSTDP Expands the Stable Learning Region in the E-I Parameter Space

The 12×12 parameter grid scan revealed that, under eSTDP only, the stable learning zone is confined to a narrow diagonal band in the E-I parameter space, indicating high sensitivity to the balance between excitatory and inhibitory drive. The introduction of iSTDP expanded this stable region, partially suppressing hyperexcitation in high-excitation conditions and preserving low-level activity in near-silent low-excitation conditions. These findings are in quantitative agreement with the analytical results of Luz and Shamir [19], who showed that Hebbian inhibitory plasticity enlarges the basin of stability for excitatory STDP, and complement their single-layer analysis by demonstrating that the same stabilizing effect generalizes to multilayer feedforward architectures.

The representative parameter trajectory revealed an approximately monotonic decline of the E/I weight ratio throughout training, driven by the progressive increase of iSTDP-regulated FS→RS inhibitory weights. This sustained co-evolution indicates that iSTDP continuously tracks excitatory activity and adjusts inhibitory strength accordingly, without exhibiting marked interference with ongoing eSTDP-mediated weight reorganization. The gradual convergence of both weight classes toward a new relative equilibrium suggests that eSTDP and iSTDP jointly approach a stable operating point, consistent with theoretical predictions of co-dependent excitatory–inhibitory plasticity [16].

It is instructive to situate the iSTDP convergence dynamics within the broader context of adaptive learning architectures. The T3-ANFIS framework recently proposed by Mohammadzadeh et al. [6] represents a notable advance in this direction, introducing a noniterative closed-form learning scheme for Type-3 fuzzy inference systems that enables efficient online adaptation of rule parameters and membership functions without iterative weight updates. The approach is particularly noteworthy for its robustness against non-Gaussian and impulsive noise and its broad applicability to real-time problems. The iSTDP rule studied here and the noniterative scheme of T3-ANFIS represent complementary solutions to the shared challenge of stable adaptive weight regulation: T3-ANFIS achieves this through closed-form adaptation laws, whereas iSTDP relies on biologically grounded Hebbian updates driven by spike timing. Taken together, these two lines of work highlight the importance of adaptive regulatory mechanisms for system stability during unsupervised learning, and suggest that cross-disciplinary exchange between biological plasticity research and engineering-oriented adaptive systems may further advance neuromorphic computing design.

### 4.3. Mechanisms Underlying Sparse Coding Formation

The controlled dissection of RS firing rate adaptation and FS lateral inhibition revealed that adaptation is the primary driver of Lifetime Sparseness ($S_{L}=0.64$ with adaptation alone vs. $S_{L}=0.15$ with FS inhibition alone), while FS inhibition plays a cooperative enhancing role. This result is consistent with the well-established view that neural adaptation promotes stimulus selectivity by compressing the dynamic range of responses [25], and provides a mechanistic account of why adaptation-equipped RS neurons naturally develop sparse representations under STDP training. The relatively modest additional gain in $S_{L}$ conferred by FS inhibition ($S_{L}=0.67$ in the complete model) suggests that lateral inhibition acts primarily as a sharpening mechanism, amplifying selectivity differences already established by adaptation rather than creating new selectivity de novo. This interpretation aligns with the role attributed to inhibitory interneurons in decorrelating cortical population responses [17].

The layer-wise sparsification profile—low at L1, rapid increase to a plateau at L2, maintained through L8—suggests that the most critical stage of sparse code formation occurs at the first feedforward synaptic transformation. The rapid $S_{L}$ increase from L1 to L2 is consistent with the hypothesis that STDP-trained feedforward synapses act as selective filters, passing activity that is strongly correlated with recurring input structure while attenuating uncorrelated activity [11]. The stability of $S_{L}$ across L2–L8 indicates that, once formed, the sparse representational structure is robustly propagated through the network, a property desirable for reliable deep-layer feature encoding. The contrasting behavior of $S_{P}$, which peaks at L2 and declines in deeper layers, may reflect progressive synchronization of deep-layer RS populations, a phenomenon that warrants further investigation in future work.

### 4.4. Temporal Pattern Selectivity and Information Preservation by Intra-Layer FS Circuits

The marked enhancement of temporal pattern selectivity by intra-layer FS connections—with the final $d^{\prime }$ of the complete model approximately 1.90 times that of the no-FS condition after training—provides direct evidence that E-I local circuits play a functional role in shaping STDP-driven temporal learning beyond their stabilizing effect on synaptic weights. The proposed mechanism—FS-mediated WTA competition concentrating eSTDP-induced potentiation onto the target-pattern-specific neuronal subpopulation—is conceptually related to the assembly formation framework of Litwin-Kumar and Doiron [30], who demonstrated that inhibitory plasticity promotes the selective consolidation of neuronal assemblies encoding specific input patterns. Our results extend this framework to a structured temporal pattern learning scenario and demonstrate that even fixed (non-plastic) intra-layer FS connections are sufficient to substantially improve temporal discrimination, suggesting that the structural presence of inhibitory interneurons, independent of their plasticity, contributes meaningfully to network selectivity.

The interval sensitivity analysis further demonstrated that the advantage of intra-layer FS connectivity is robust across a range of biologically plausible inter-group delays (3–8 ms), with the complete model maintaining $d^{\prime }>1$ at 3 and 5 ms and approaching the threshold at 8 ms, while the no-FS condition remained below the threshold at all tested intervals. The breakdown of reliable discrimination at intervals exceeding 8 ms in both conditions is consistent with the temporal integration window of the eSTDP rule ($\tau_{\pm }=20$ ms), beyond which sequential spike pairs fall outside the effective potentiation window. These results confirm that the 5 ms interval used in the main experiment lies within the optimal operating range of the network.

The pattern generalization test further revealed that the network acquired selectivity primarily for the temporal order structure of the input sequence rather than the identities of the activated neurons. Responses were well preserved under small temporal jitter (±2 ms) but were substantially reduced when the sequential order was disrupted or the neuron groupings were randomized. This order-specificity is consistent with the spike-timing-dependent nature of the underlying plasticity rule, in which the precise sequence of pre- and postsynaptic firing determines the sign and magnitude of synaptic modification.

The mutual information analysis showed that the complete model consistently maintained higher I(S;R) than the no-FS condition across all deep layers (L2–L8), with the difference most apparent near L3 (approximately 2.2 bits vs. 1.85 bits). Although individual layer-wise comparisons did not reach statistical significance (all p>0.05), likely due to limited statistical power with five independent runs, the consistent direction of the effect across all deep layers is indicative of a systematic tendency for intra-layer FS circuits to preserve more stimulus-related information during feedforward propagation, a conclusion further supported by the significant improvement in $d^{\prime }$ observed in the complete model. From a neuromorphic computing perspective, this finding suggests that embedding inhibitory interneuron circuits within each processing layer of a deep SNN architecture can provide sustained benefits for information fidelity, a design principle that may be particularly relevant for event-driven hardware implementations where maintaining sparse, stimulus-specific activity patterns is essential for energy efficiency [4].

### 4.5. Limitations and Future Directions

Several limitations of the present study should be acknowledged. First, the network architecture is restricted to a strictly feedforward topology, lacking top-down feedback connections that are prominent in biological cortical hierarchies. Incorporating recurrent inter-layer connectivity may reveal additional dynamics not captured by the present model. Second, the present study examined a single fixed network depth; ablation studies varying the number of layers would help clarify how hierarchical depth influences E-I cooperative dynamics and pattern completion performance. Third, all simulations were conducted in a rate-homogeneous input regime using Poisson spike trains; naturalistic stimuli with complex spatiotemporal structure may engage E-I cooperative mechanisms differently, and evaluating the model under such conditions represents an important direction for future work. Fourth, although robustness across inter-group intervals and pattern types was evaluated, the present study examined a single fixed neuron grouping and a single target pattern; extending the analysis to multiple target patterns and more complex spatiotemporal structures would further clarify the generalization capacity of the network. Fifth, the present study focuses on a single representative parameter configuration for the detailed trajectory analysis; a more comprehensive exploration of the iSTDP learning rate space and weight bound settings would further clarify the robustness of the cooperative convergence dynamics. Sixth, although the proposed network provides bioinspired design principles for neuromorphic systems, the model has not been validated on neuromorphic hardware platforms such as Intel Loihi. Mapping the present E-I circuit architecture onto event-driven hardware and evaluating energy efficiency under sparse coding constraints constitutes a natural next step toward practical neuromorphic implementation.

## 5. Conclusions

In this study, we constructed an eight-layer feedforward RS-FS spiking neural network incorporating intra-layer E-I connectivity and systematically examined the multilayer learning dynamics under the cooperative action of eSTDP and iSTDP. The regulatory role of intra-layer E-I circuits was analyzed across six dimensions: synaptic weight evolution, E-I parameter stability, sparse coding formation, temporal pattern selective learning, inter-group interval sensitivity, and pattern generalization.

At the level of synaptic learning dynamics, intra-layer FS lateral inhibition was shown to alleviate the LTD bias induced by RS firing rate adaptation through a WTA competition mechanism, promoting heterogeneous differentiation of E→E weights while maintaining stable mean synaptic strength. Compared with the condition without intra-layer connectivity, the E→E weight variance under the complete model increased approximately 3.36-fold, demonstrating that intra-layer E-I circuits primarily influence feedforward weight reorganization by enhancing competitive differentiation within the synaptic population rather than by altering mean synaptic strength.

At the level of network stability, the 12×12 parameter grid scan demonstrated that the stable learning zone under eSTDP only is largely confined to a narrow range of E-I parameter combinations, with the network exhibiting high sensitivity to intra-layer excitatory and inhibitory strengths. The introduction of iSTDP expanded the region of the parameter space supporting effective learning, partially alleviating the hyperexcitation and high-variance states in high-excitation, low-inhibition conditions, and enabling the network to maintain low but non-zero RS population activity in some low-excitation parameter regions. The temporal evolution under representative parameters further showed that the mean E→E weight remained relatively stable throughout training while the iSTDP-regulated FS→RS inhibitory weight increased monotonically, transitioning the E/I weight relationship to a new relative equilibrium. This indicates that eSTDP and iSTDP can achieve cooperative regulation in this network, improving adaptability to moderate E-I parameter mismatch.

At the level of sparse coding, the comparison of three control conditions demonstrated that RS firing rate adaptation is the primary factor promoting Lifetime Sparseness formation ($S_{L}=0.64$ with adaptation alone; $S_{L}=0.15$ with FS inhibition alone; $S_{L}=0.67$ in the complete model), while FS lateral inhibition exerts a cooperative enhancing effect through WTA competition. Population Sparseness showed small differences across conditions (approximately 0.05–0.09), suggesting that $S_{P}$ is less sensitive to these two mechanisms and may more strongly reflect the overall network activity state and population synchrony. Layer-wise analysis further revealed that $S_{L}$ rapidly increased from approximately 0.25 at L1 to approximately 0.73 at L2 and was maintained at a high plateau of approximately 0.74 through L2–L8, while $S_{P}$ peaked at approximately 0.35 at L2 and gradually declined to approximately 0.02 in the deep layers. These results indicate that sparse coding is predominantly formed during L1-to-L2 propagation and is subsequently maintained in the form of high Lifetime Sparseness across deeper layers.

At the level of temporal pattern learning and information transmission, the complete RS-FS network exhibited stronger selectivity for the target temporal pattern after STDP training, with $d^{\prime }$ and AUC both improving substantially after training. The final $d^{\prime }$ of the complete model was approximately 1.90 times that of the no-FS condition (Welch’s *t*-test: p<0.001), demonstrating that intra-layer FS lateral inhibition contributes to enhancing temporal pattern selective learning. The interval sensitivity analysis confirmed that this advantage is robust across inter-group delays of 3–8 ms, with reliable discrimination ($d^{\prime }>1$) achieved by the complete model at 3 and 5 ms but not by the no-FS condition at any tested interval. The pattern generalization test further showed that the network acquired selectivity for the temporal order structure of the input sequence: responses were well preserved under small temporal jitter (±2 ms) but substantially reduced when the sequential order was disrupted or neuron groupings were randomized. Mutual information analysis revealed a consistent tendency for the complete model to maintain higher I(S;R) than the no-FS condition across deep layers (L2–L8), most apparent near L3, although individual layer-wise differences did not reach statistical significance (p>0.05), likely reflecting limited statistical power with five independent runs. Taken together, these results indicate that intra-layer FS circuits contribute to enhancing both the discriminability of stimulus-related responses and the preservation of information during deep-layer feedforward propagation.

In summary, intra-layer E-I circuits play important regulatory roles in synaptic weight differentiation, parameter stability, sparse coding formation, and temporal pattern selectivity in multilayer feedforward STDP learning. The cooperative action of eSTDP and iSTDP not only contributes to maintaining a relative balance between excitatory and inhibitory synaptic weights but also promotes the formation of selective representations and temporal information processing capacity in multilayer spiking networks. The robustness of these effects across inter-group intervals and pattern types further supports the functional generality of the proposed E-I cooperative learning framework. Within the scope of the present eight-layer feedforward model, these findings provide computational model support for understanding cortical E-I cooperative plasticity mechanisms and offer bioinspired references for the design of neuromorphic systems with adaptive inhibitory regulation capability.

## Data Availability

The original contributions presented in the study are included in the article, further inquiries can be directed to the corresponding author.

## Abbreviations

The following abbreviations are used in this manuscript:

AUC	Area under the curve
E-I	Excitatory–inhibitory
eSTDP	Excitatory spike-timing-dependent plasticity
FS	Fast spiking
iSTDP	Inhibitory spike-timing-dependent plasticity
LTD	Long-term depression
LTP	Long-term potentiation
RS	Regular spiking
ROC	Receiver operating characteristic
SNN	Spiking neural network
$S_{L}$	Lifetime Sparseness
$S_{P}$	Population Sparseness
STDP	Spike-timing-dependent plasticity
WTA	Winner-take-all
